# Rapid Cohort Generation and Analysis of Disease Spectrum of Large Animal Model of Cone Dystrophy

**DOI:** 10.1371/journal.pone.0071363

**Published:** 2013-08-19

**Authors:** Corinne Kostic, Simon Geoffrey Lillico, Sylvain Vincent Crippa, Nicolas Grandchamp, Héloïse Pilet, Stéphanie Philippe, Zen Lu, Tim James King, Jacques Mallet, Chamsy Sarkis, Yvan Arsenijevic, Christopher Bruce Alexander Whitelaw

**Affiliations:** 1 Unit of Gene Therapy and Stem Cell Biology, Jules-Gonin Eye Hospital, University of Lausanne, Lausanne, Switzerland; 2 The Roslin Institute and R(D)SVS, University of Edinburgh, Easter Bush Campus, Midlothian, United Kingdom; 3 Unit of Biotechnology and Biotherapy, Centre de recherche de l’Institut du Cerveau et de la Moelle Epinière, Pierre-and-Marie-Curie University/Institut National de la Santé et de la Recherche Médicale/UMR_S975/CNRS UMR7225, Paris, France; 4 NewVectys, Villebon-sur-Yvette, France; Justus-Liebig-University Giessen, Germany

## Abstract

Large animal models are an important resource for the understanding of human disease and for evaluating the applicability of new therapies to human patients. For many diseases, such as cone dystrophy, research effort is hampered by the lack of such models. Lentiviral transgenesis is a methodology broadly applicable to animals from many different species. When conjugated to the expression of a dominant mutant protein, this technology offers an attractive approach to generate new large animal models in a heterogeneous background. We adopted this strategy to mimic the phenotype diversity encounter in humans and generate a cohort of pigs for cone dystrophy by expressing a dominant mutant allele of the guanylate cyclase 2D (GUCY2D) gene. Sixty percent of the piglets were transgenic, with mutant GUCY2D mRNA detected in the retina of all animals tested. Functional impairment of vision was observed among the transgenic pigs at 3 months of age, with a follow-up at 1 year indicating a subsequent slower progression of phenotype. Abnormal retina morphology, notably among the cone photoreceptor cell population, was observed exclusively amongst the transgenic animals. Of particular note, these transgenic animals were characterized by a range in the severity of the phenotype, reflecting the human clinical situation. We demonstrate that a transgenic approach using lentiviral vectors offers a powerful tool for large animal model development. Not only is the efficiency of transgenesis higher than conventional transgenic methodology but this technique also produces a heterogeneous cohort of transgenic animals that mimics the genetic variation encountered in human patients.

## Introduction

Current preclinical trials are heavily reliant on rodents, although their usefulness as disease models may be restricted because of major differences in body size, general physiology, anatomy, diet or lifespan. Thus, replication of a human mutation in mice can result in a significantly different disease phenotype, as exemplified in the retinoblastoma [1] or cystic fibrosis [2] transgenic mouse models. It is recognized that the efficacy of translation of new drugs or techniques to the clinic would benefit from evaluation and testing in large animals, which have organ size and metabolism similar to humans [3]. There is therefore a need to extend genetically-defined disease models beyond mice to other species.

Somatic cell nuclear transfer (SCNT) from transgenic donor cells has been widely used as a method for pig transgenesis, but is technically demanding, time consuming, and typically suffers from small litter sizes. Furthermore, given that the cells used for SCNT are typically clonal the resultant cohort of animals lacks the heterogeneity of an outbred population; this can have disadvantages when modeling a disease with heterogeneous phenotype. As an alternative strategy, transgenic animals have been produced with high efficiency using lentiviral vectors such as the human immunodeficiency virus type 1 (HIV-1), simian immunodeficiency virus (SIV) and equine infectious anemia virus (EIAV) derived vectors [4]–[7]. This technology has been effectively applied to many different species including, rats, rabbits, pigs, chicken, sheep and non-human primates [8]–[12], mostly to create green fluorescent protein (GFP) transgenic animals and in few cases to model human disease. Lentiviral transgenesis may be applied to generate animal models by overexpression of a dominant negative mutant allele. This method should give a broader picture of the disease state than can be achieved by SCNT in that each individual in the cohort will be genetically unique, both base genetics and expression profile from multiple transgene insertion sites. This heterogeneity should thus permit modeling of the range of phenotypes observed in human patients affected by a disease and displaying heterogeneity of penetrance or severity.

Retinal diseases displays such heterogeneity of penetrance and severity, and while retinal function and morphology can be easily evaluated *in vivo* allowing effective follow up in live animals, there is a real need for retinal disease models for biomedical research as the rodent retina poorly reproduces human retina, especially regarding cone content. Rodents have a retinal anatomy very different from humans. The mouse neuroretina is composed mainly of rod photoreceptors, with cones constituting only 3% of the total photoreceptor population. The mouse retina is thus similar to the human peripheral retina. Cones in human are concentrated in the macula (the central region of the retina) and exclusively compose the fovea. This central region is essential for sharp vision in conditions of bright light, with the surrounding rod-enriched peripheral zone providing greater sensitivity on lower light conditions. While the mouse can provide a good model for diseases affecting the peripheral retina or rods, its anatomy renders it inappropriate for models of cone-dystrophies and macular degeneration [13]. Naturally occurring canine retinal models exist and these often have advantages over murine transgenic models: more progressive degeneration mimicking more closely the human pathology and eye size closer to the humans’ allowing the efficiency of therapies to be tested at an appropriate surgical scale. However, the number of dog colonies established for preclinical research is very limited. Indeed, such colonies mostly rely on fortuitous discovery and require prolonged breeding strategies to generate experimental cohorts. In addition, identified mutations may be associated with additional disease phenotype, thus rendering precise analysis difficult. As a consequence, dog models are unpredictable and costly [14], [15].

The pig is an appropriate alternative choice as an ophthalmology model since it has an enriched cone region in the central part of the retina making a transversal naso-temporal streak resembling the primate macula. Moreover, as pig can be genetically engineered with relative ease this species is an attractive alternative for *de novo* model generation.

In the current study we investigated whether lentiviral transgenesis could be applied to rapidly produce a heterogeneous cohort of transgenic animals bearing different copy numbers of a dominant-negative transgene inducing cone(-rod) dystrophy (CORD). CORDs represent around 10–20% of all retinal dystrophies and are genetically and phenotypically heterogeneous. The loss of vision is progressive with first report of visual acuity impairment in the first decade, followed by nyctalopia, decrease of peripheral vision and loss of color vision. On the histological level, structural alteration of outer-segments and reduction of the retina thickness have been reported. Although genetic studies in humans have associated several loci to CORD, how specific mutations affect the cone cells is until now poorly understood in part due to the lack of relevant models.

The GUCY2D gene codes for a guanylate cyclase, an enzyme involved in the regulation of calcium influx in rods and cones. Mutations in this gene, identified as the CORD6 locus, show the most prevalent association with dominant transmission of the CORD [16]–[18]. GUCY2D activity indirectly depends on intracellular calcium levels via activation and binding of the guanylate cyclase activating protein (GCAP). Recent reports suggest that dominant mutations of GUCY2D increase the stability of the protein which favors the binding of the GCAP independently of its activation status [19]. The activation of the GUCY2D mutant would thus be less sensitive to the intracellular calcium level, and would lead to excess of intracellular calcium in photoreceptors, as suggested by *in vitro* studies [20], [21]. The GUCY2D gene is expressed both in rods and cones and the reason why this mutation primarily affects cones is poorly understood. This question can hardly be elucidated in rodent models, we thus generated transgenic pigs expressing the double mutant GUCY2D^E837D/R838S^. Visual tests and histological analyses performed on transgenic animals show heterogeneous phenotypes with common alterations including decreased cone function and disorganization of cones consistent with clinical reports [18]. The CORD6 pig model generated in this study thus represent a valuable animal model for translational studies. Moreover, the transgenic methodology can be applied to other diseases where heterogeneity of phenotypes is sought.

## Methods

### Ethical Statement

The animals were handled in accordance with the statement of the “Animals in Research Committee” of the Association for Research in Vision and Ophthalmology and with appropriate authorized and licencing under the Animal (Scientific Procedures) Act 1986 in the UK. The work carried out in this study was given approval by the Roslin Institute Animal Welfare and Ethics Committee.

### Comparative Promoter Analysis

**Figure 1:**
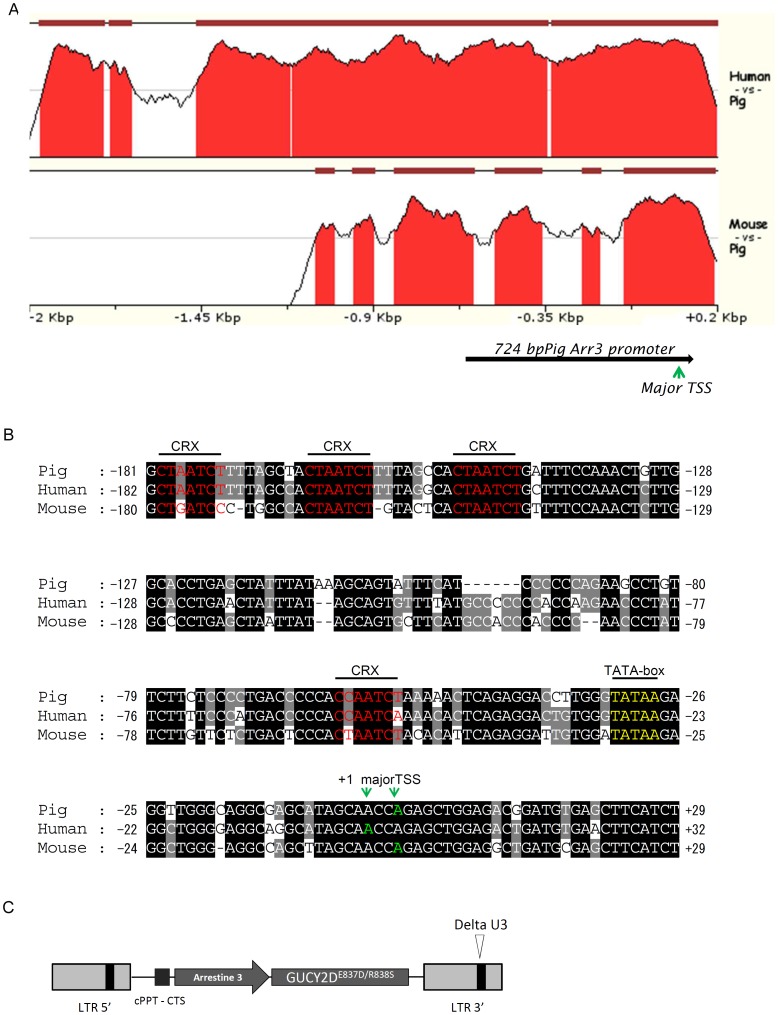
Strategy for the GUCY2D^E837D/R838S^ transgenesis. (A) Comparative analysis of the cone arrestin promoters from human, pig and mouse was performed. The 2 kbp upstream plus 200 bp downstream human and pig Arr3 promoters were aligned against that of the pig. Brown Conserved blocks with similarity higher than 70% were colored red under the curve. (B) The core promoter region was found to be highly conserved in pig, human and mouse. Four potential binding sites for the CRX photoreceptor transcription factor could be identified while the conserved TATA-boxes are located about −30 bp upstream of the published transcriptional start sites (TSS) [33], [60]. TSS of the pig *Arr3* gene was predicted by comparing the sequence with the JASPAR’s initiator weight matrix. Other promoter elements, such as CAAT-box, SP1, TATA-box associated with minor TSS, were not shown. (C) Schematic of the transgene cassette encoding the human mutant GUCY2D^E837D/R838S^ cDNA under the control of a fragment of the pig cone arrestin promoter. LTR: Long terminal repeat, the 3′ LTR is designated as “delta U3” for a fragment of the U3 region has been deleted, rendering the vector “self-inactivating”, i.e. unable to replicate once in the target cell, this also prevents interference of the HIV promoter with the internal Arr3 promoter; cPPT-CTS: central polypurine tract – Central termination site.

The 2 kbp upstream plus 200 bp downstream genomic regions flanking the respective bp one of the pig, human and mouse *Arr3* reference mRNA (NM_214345, NM_004312 and NM_133205) were extracted for the initial promoter analysis. These promoter sequences were first aligned with Lastz (v1.03.02) [22], allowing 8 mismatches out of the 19 bp seed, and blocks of conserved regions detected in 50 bp sliding windows were visualized with zPicture [23] (Fig. 1*C*). Transcription factor binding sites falling on these regions were scanned using the MATCH algorithm included in TRANSFAC 7.0 [24] while those on the core promoter regions were searched against the JASPAR’s POLII sub-database (http://jaspar.genereg.net/). Refined alignment (Fig. 1*D*) of the core promoter region of the 3 sequences was done using the program MUSCLE (v3.8.31) [25].

### Transgenesis and Genotyping

Transgenic pigs were generated and genotyped as described previously [7]. Briefly, zygotes were collected by flushing the oviducts of artificially-inseminated super-ovulated Large-White gilts. 50 or 500 pl lentivirus suspension (148 ng p24/µL) was injected into the perivitelline space, following which 30 zygotes were transferred to the oviduct of identically treated but unmated recipients. Genomic DNA was prepared from ear biopsies, and initially assessed for presence of lentivirus-vector transgene integrants by PCR with primers HIV1 (GAGAGAGATGGGTGCGAGAG) and HIV2 (GCTGTGCGGTGGTCTTACTT) which span the lentiviral packaging signal sequence, and the transgene copy-number subsequently determined by Southern blot.

### RT-PCR

Retina RNA extraction was performed in Trizol (Life Technologies) according to manufacturer instructions and following physical disruption. Total RNA were purified using RNeasy Mini Kit (Qiagen). Reverse transcription was performed using SUPERSCRIPT 3 1^st^ STRAND (Invitrogen) and oligo dT. cDNAs were then amplified with specific primers: pig glyceraldehyde-3-phosphate dehydrogenase (GAPDH) cDNA (GATGGTGAAGGTCGGAGTGA and AGGCATTGCTGACGATCTTG), pig GUCY2D cDNA (GAGGACCTGATCGGGGAGC and CACCTTGTAGACATCATGGGAG), human mutant GUCY2D cDNA (GAGGATCTGATCCGGGACA and TCTCCACCTTGTAGACATCG).

### Full-field Electroretinogram (ERG)

Animals were sedated with ketamine (10 mg/kg, intramuscular injection, Vetalar V, Pfizer Ltd) and azaperone (2 mg/kg, intramuscular injection, Stresnil, Elanco Animal Health) and maintained anesthetized under oxygen, by intravenous injection of a mix of ketamine (2 mg/kg) and midazolam (0.2 mg/kg, Hypnovel, Roche Products Ltd). Pupils were dilated by topical administration Tropicamide 0.5% (Mydriacyl Eye Drops, Alcon), and phenylephrine 10% (phenylephrine hydrochloride eye drops, Bausch and Lomb). Unilateral recording was performed using DTL electrodes and the RETI-com portable device (Roland Consult, Brandenburg an der Havel). After 5 to 10 minutes opened eyelids under room light, a photopic protocol described by International Society for Clinical Electrophysiology of Vision (ISCEV) [26] was used to measure single flash and 30 Hz flicker at 3 cds/m^2^ and a supplementary single flash at 10 cds/m^2^ and 10 Hz flicker at 3 cds/m^2^. The response was established by averaging 8 flashes of 5 s interval for the 3 cds/m^2^ stimuli and 4 flashes of 5 s interval for the 10 cds/m^2^ stimuli and 8 flashes for flickers.

### Behavioural Test

At 11 weeks of age a maze was constructed with 2 panels where a passage was opened in order to let the animals go through it to reach a color object (yellow and blue) laying at the end. The luminance varied from 500 to 800 lux through the maze. The animals were recorded and the time to reach the object was quantified.

At 24 and 52 weeks, obstacle courses were arranged in corridors of 1.32 and 2.38 m wide and 10 and 13 m long respectively. Red construction cones (47 and 77 cm of height respectively) were used as main obstacles. Additional obstacles were used for the 24 weeks test: some buckets (27 to 33 cm of diameter and 21 to 25 cm of height) and a Frisbee hanged up at 47 cm high. The luminance varied from 1000 to 2000 lux for the 24 week test and from 800 to 1300 lux for the 52 week test, all above 500 lux to ensure minimal rod input. The animals were recorded during their progression through the obstacles and then the videos analyzed to determine the time for reaching the end of the course, the number of errors (unintentional contact with an obstacle as evidenced by a startle response to the contact) and the number of objects that were approached by other senses than vision like sniffing or licking, a behavior we called “alternative prospection” which would suggest that vision is not sufficient for them to appraise their environment. We also determined a score as the sum of errors or alternative prospection for each pig.

### *In vivo* Imaging

Retinal morphology in the region spanning 1.5 to 4 mm dorsal to the optic nerve was assessed under anesthesia using the spectral domain optical coherence tomography (SDOCT) from Bioptigen (Durham).

### Histology-Immunohistology

Collected eyes were superficially cauterized, incubated overnight (4°C) in 4% paraformaldehyde and stored in PBS until processing. The eye were orientated and cut vertically in half: the nasal hemisphere was equilibrated in PBS with 30% sucrose before embedding in home-made mounting medium (albumin from hen egg white (Fluka) mixed with gelatin) for cryosection. The temporal hemisphere was paraffin-embedded. Cryosections of 16 µm and microtome paraffin sections of 6 µm were collected on superfrost plus slides (Menzel-Gläzer) and stored at −20°C.

For immunohistological analysis on cryosections, slides were rehydrated and blocked for 1 hour at room temperature in PBS with 10% normal goat serum (Dako) or donkey serum (Milan Analytica AG) and 0.2% Triton-X 100. Peanut agglutinin lectin (lectin from arachis hypogaea, PNA) labeling was performed using PNA-TRITC (1∶4000, Sigma). Primary antibodies: medium wavelength opsin (M-opsin) rabbit antibody (1∶1000, Chemicon), short wavelength opsin (S-opsin) goat antibody (1∶1000, Santa Cruz), glial fibrillary acidic protein (GFAP) rabbit antibody (1∶400, Dako). Secondary antibodies (1∶2000, Alexa FLuor©488 goat anti-rabbit IgG or Alexa Fluor©488 donkey anti-goat IgG, Invitrogen). DAPI (4′,6′-diamidino-2-phenylindole, Molecular Probes inc) counterstaining was performed and the sections were mounted with Mowiol ©4–88 (VWR). Paraffin sections were stained with the standard hematoxylin-eosin (HE) staining.

### IHC Quantification

Central sections along the naso-temporal axis were used for quantification. Displaced nuclei were determined in the full section stained by hematoxylin-eosin. Counting of PNA or M-opsin outersegments was performed across a 100 to 300 µm length of the retina, 1.6 mm superior to the optic nerve, in the visual streak as described by Hendrickson *et al.* [27].

### Statistical Analysis

Statistical analyses of the electroretinograms and histological data were performed by two way-ANOVA and post-tests determined the statistical significance between the different groups (transgenic and non-transgenic).

## Results

### Strategy to Engineer a Cone Dystrophic Model in Pig

Modelling cone dystrophy in a large animal is of prime importance to understand cone pathophysiology and to test new treatments in a physiologically relevant context. Among large animal species, swine is particularly relevant to study cones because of its similarity in size and metabolism to human and because it has an enriched region for cones in its retina. Additionally, pig transgenesis is efficiently performed using a lentiviral vector as we previously showed [7], reaching efficiency rates typically between 20 to 100 per cent. Lentiviral transgenesis can be used to specifically express a dominant mutant allele, thereby inducing pathology in the transgenic animals. Moreover, as each transgenic animal of the F0 generation (founder population) is unique, being derived from individually transduced zygotes, we should obtain a cohort of transgenic animals with different transgene copy numbers and genome integration sites which will result in a range of transgene expression profiles and thus a range of pathology severity. The overall strategy is thus to construct a lentiviral vector for expression of a dominant mutated cDNA driven by a cone-specific promoter and to use this vector to generate a heterogeneous cohort of transgenic pigs and finally to assess the pathological features of the candidate mutation.

Some mutations of the GUCY2D gene are identified as a major cause of autosomal dominant inherited cone degeneration in humans [28], such as CORD6 dystrophy where cones and subsequently rods are affected. The GUCY2D^E837D/R838S^ double mutant allele was used in this study because this mutant is linked to a more severe phenotype than single mutants. In order to generate animals with potential cone degeneration, we designed a vector where transcription of the mutant allele is controlled by a fragment of a cone specific promoter. The cone arrestin-3 promoter demonstrated cone-specific expression in Xenopus and mouse [29]–[31]. In addition, a transcriptomic study of 35 different pig tissues found the arrestin-3 gene to be expressed only in the retina [32]. To further ensure this specific regulation in pig, we aligned the 2.2 kbp human and mouse cone arrestin promoter region against that of the pig.

The pig *Arr3* promoter shares significant conserved regions with that of human and mouse in the −1.2 kbp and the core promoter regions (Figure 1A). *Cis*-elements essential for transcription initiation, such as TATA-box and *Inr*, and the binding sites for the photoreceptor transcription factor CRX are conserved in all three promoters (Figure 1B). To avoid the potential negative regulation on the promoter activity observed by Zhu *et al*. [33], a search for repressor binding sites was carried out on the three promoters. A conserved binding site for the CCAAT-box displacement repressor protein was identified at about 760 bp upstream of the pig transcription start site. We thus selected a 724 bp pig arrestin-3 promoter made up of a sequence from −687 to +36 bp of transcription start site. The designed pig arrestin-3 promoter was inserted into a lentiviral vector backbone upstream of the cDNA of the human GUCY2D gene containing 2 substitutions 837 E->D and 838 R->S (Figure 1C).

### Efficient Lentiviral GUCY2D^E837D/R838S^ Transgenesis in Pig

**Figure 2:**
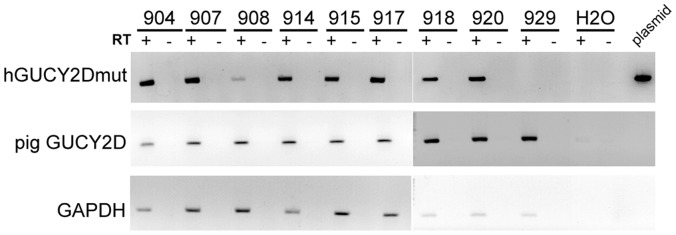
Expression of GUCY2D^E837D/R838S^ transcript in transgenic pigs. Assessment of transgene expression by RT-PCR. Transgenic pigs: 904, 907, 908, 914, 915, 917, 918, 920, control non-transgenic pig (929). +: with reverse transcription; −: without reverse transcription; hGUCY2Dmut: GUCY2D^E837D/R838S^ PCR fragment; pig GUCY2D: pig GUCY2D PCR fragment; GAPDH: pig GAPDH PCR fragment.

Using the transgene cassette described above, lentiviral vectors were produced and transgenic pigs were generated as described previously [7]. From two recipient animals, 22 piglets were born, of which 13 were confirmed as transgenic by Southern blot (Table 1) representing a 60% transgenesis efficiency. Seven non-transgenic piglets were examined in this study as controls. On a gross level transgenic offspring were indistinguishable from controls both physiologically and behaviourally. Animals were co-housed and cared for using standard husbandry practice. To assess for transgene expression, we performed RT-PCR on RNA extracts from the retina of the right eyes of 8 transgenic pigs sacrificed at 78 weeks. The GUCY2D^E837D/R838S^ transcript was found in all transgenic pigs (904, 907, 908, 914, 915, 917, 918, 920) but not in control animal (929) while PCR for the endogenous pig GUCY2D mRNA detected transcript in both transgenic and control animals (929) (Table 1, Figure 2). These results taken together confirm the efficiency of lentiviral-mediated transgenesis to generate pigs expressing the human GUCY2D^E837D/R838S^ allele in their retina.

**Table 1:** Identification of the number of copy and the retinal expression of the transgene in GUCY2D^E837D/R838S^ transgenic pigs.

sow ID	piglet ID	copy No.	sex	RT-PCR
**4023**	**902**	3	m	nd
	**904**	1	m	+
	**906**	1	m	+
	**907**	5	m	+
	**908**	2	m	+
	**909**	3	f	nd
**4020**	**913**	5	m	nd
	**914**	2	m	+
	**915**	2	m	+
	**917**	3	m	+
	**918**	3	m	+
	**919**	6	m	nd
	**920**	4	m	+

ID: identification of the animal; No: number; RT-PCR; amplification of GUCY2D^E837D/R838S^ transcript; m: male; f: female; +: amplification of the fragment of GUCY2D^E837D/R838S^ transcript; −: no amplification of the fragment of GUCY2D^E837D/R838S^ transcript; nd: not determined.

### Impairment of Retinal Function in GUCY2D^E837D/R838S^ Transgenic Pigs: Electrophysiological Assessment

**Figure 3:**
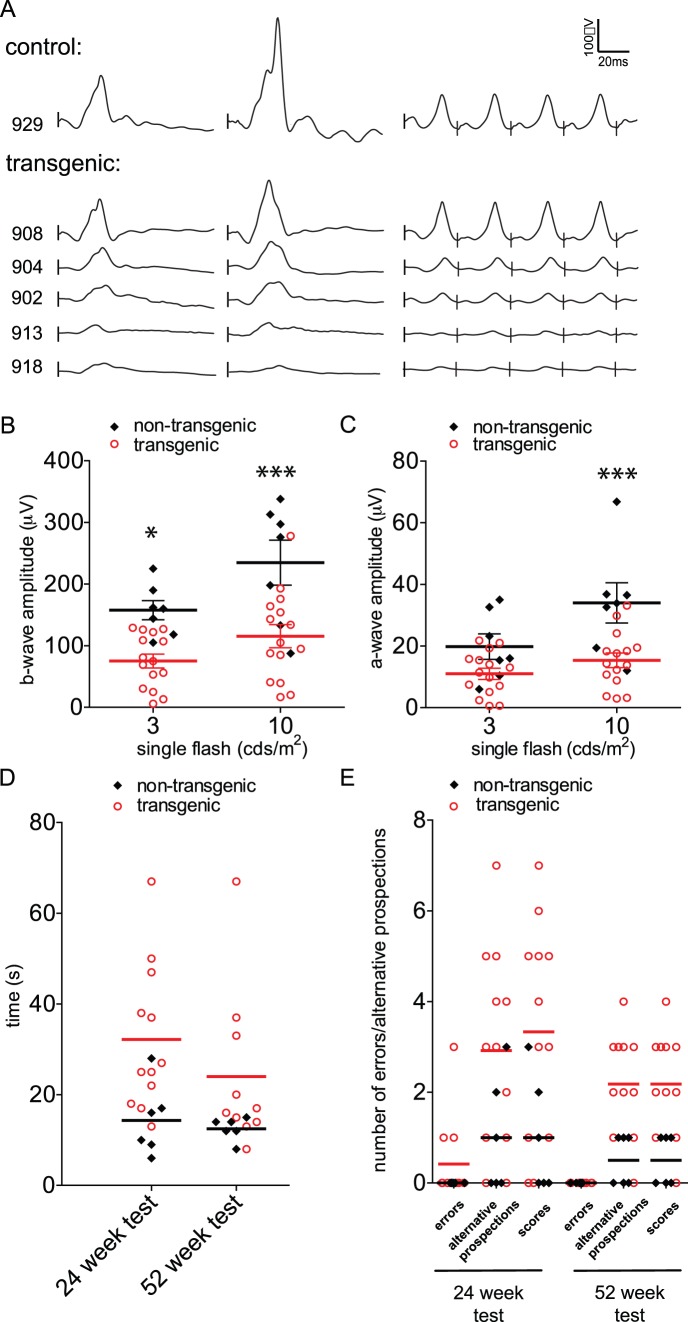
Range of visual function in GUCY2D^E837D/R838S^ transgenic pigs. (A) Traces of photopic electroretinogram recordings (ERG) at 11 weeks are shown for one representative control and several transgenic animals. (A, first column) single flash at 3 cds/m^2^, (A, second column) single flash at 10 cds/m2, (A, third column) flickers at 3 cds/m^2^,30 Hz. (B) b-wave amplitudes for all examined animals at 11 weeks with single flash at 3 cds/m^2^ and at 10 cds/m^2^. (C) a-wave amplitudes obtained for all examined animals at 11 weeks with single flash at 3 cds/m^2^ and at 10 cds/m^2^. (D) Representation of the time needed to complete the obstacle course at 24 and 52 weeks for transgenic and non-transgenic control animals. (E) Representation of the errors (missing or striking into an obstacle), alternative prospections (the number of times individuals investigated an obstacle by sniffing or licking) and resulting scores from the obstacle course at 24 and 52 weeks for transgenic and non-transgenic control animals. Horizontal bars in B,C represent the mean of the different groups with the SEM; *: p<0.05;***: p<0.001; errors in E: miss or strike into an obstacles; alternative prospection in E: sniff or lick the obstacles; score in E: sum of errors and alternative prospections.

In order to determine whether the expression of the dominant negative mutant GUCY2D^E837D/R838S^ induces a phenotype, the GUCY2D^E837D/R838S^ transgenic pigs were first characterized by functional analysis of the retina. The retina activity under photopic (well lit) conditions, which originates mainly from the cones, was measured by electroretinogram (ERG) recording at 11 and 52 weeks of age, with a protocol used for human examination [26]. A reduction of the amplitude was observed in transgenic group, although the kinetics of the response was well preserved compared to control animals. This decrease was observed for both single flash and flicker stimuli, the latter revealing more specifically cone activity (Figure 3A, third column) and this phenotype was observed at both ages assessed (Figure 3B, 3C). Despite heterogeneity, the difference of amplitude between transgenic and control groups was statistically significant at 11 weeks, at both single flash intensities for the b-wave but only at higher intensity for the a-wave (*p = 0.04*, Figure 3B,C). Decreased a-wave reveals partial loss of photoreceptor activity, whereas the b-wave, generated by interneurons processing the output of photoreceptors, is more severely affected as it represents a signal amplification. ERG results thus highlight that GUCY2D^E837D/R838S^ overexpression negatively impacted on cone activity, with early onset in most animals and high variability among transgenic individuals.

Measurement of retinal response at 52 weeks revealed increased variation in both the control and transgenic groups with an average response lower than recorded at 11 weeks (mean b-wave amplitude ± SEM for controls: 235 µV ±40 (n = 6) at 11 weeks vs 158 µV ±38 (n = 6) at 52 weeks). Seven out of the 10 transgenic pigs examined had a b-wave amplitude lower than the mean of control animals, although results were not statistically significant. ERG analysis at this latter time point thus reveals that the alteration of the ERG response is maintained and the variability is further increased compared to analysis at 11 weeks.

### Impairment of Retinal Function in GUCY2D^E837D/R838S^ Transgenic Pigs: Behavioral Assessment

Electroretinogram recording objectively measures the global retinal activity and can be affected before the individual effectively reports visual impairment [34]. The useful vision of the animals was thus further evaluated with behavioural tests. These tests were performed at 11, 24 and 52 weeks of age and, as for ERG measurement, they were conducted under photopic conditions to minimise the input of rods. Desensitization of the rod system has been previously characterized in mice: a light intensity as low as 150 lux is sufficient to reduce rod activity to 50% of their maximal response, and rod activity is further reduced to 30% of the peak response under 1750 lux illumination [35]. In contrast to mouse, humans have a retina adapted to day activity and, as a consequence, although the saturation of human rods is still debated, the highest luminance proposed is much lower than in mouse; between 10 and 100 lux [36]. These parameters have not been evaluated in pig retina. However, because its composition and organisation are also adapted to a diurnal activity mainly using cones, we hypothesized that a light intensity above 500 lux should not allow much rod input in the general visual function. Thus visual behaviour was evaluated between 500 and 2000 lux at the 11, 24 and 52 weeks of age. To accommodate the size differences of the animals between tests (approximately 40 kg at 11 weeks vs approximately 180 kg at 52 weeks) and to minimise association, the design of the test was modified between the three time points. For this reason, results should be considered independently, providing comparison between groups within each time point and not between time points. Pictures of the 3 tests set up are presented on Figure S1. Examples of animals navigating through the obstacle course are presented in Movie S1.

Mobility at 11 weeks was evaluated by (1) measuring the time for the pig to pass through doors to reach an object, and (2) determining whether the animal bumped into the doors. At 24 and 52 weeks we quantified the success of the obstacle course based on 3 criteria: (1) the time to complete the course, (2) the number of obstacles that the animal touched (“errors”) and (3) the number of obstacles that the animal explored using other senses than vision like smelling or licking (“alternative prospections”). Combination of these 2 last criteria determined a global score.

The first test at 11 weeks of age didn’t reveal any severe visual handicap with no significant difference between transgenic and control groups on the number of errors or time to accomplish the task (non-transgenic mean time (n = 5) 5.4 s ±1.3; transgenic mean time (n = 13) 5.7±1). The absence of severe visual impairment and the decreased ERGs measured at the same time point suggest an early stage of the disease.

Differences between transgenic and control animals became apparent at 24 and 52 weeks. While the performances of the control animals are homogeneous (control mean time ± SEM at 24 weeks (n = 6) 14.3 s ±3.2, at 52 weeks (n = 6) 12.5 s ±1; mean score ± SEM at 24 weeks 1±0.5, at 52 weeks 0.5±0.2), the scores of the transgenic animals are increased and more widely scattered (transgenic mean time ± SEM at 24 weeks (n = 12) 32.2 s ±4.6, at 52 weeks (n = 10) 24 s ±5.6; mean score ± SEM at 24 weeks 3.3±0.7, at 52 weeks 2.2±0.4; Figure 3D,E). The visual impairment is not obviously detected through the “error” criteria: only 3 transgenic animals out of 12 animals analyzed at 24 weeks missed obstacles. Interestingly, the “alternative prospection” criteria revealed a particular behaviour: most of the transgenic pigs (10 out of 12 at 24 weeks and 10 out of 11 at 52 weeks) smelt or licked some obstacles before continuing the obstacle course (Figure 3E). This particular behaviour is generally associated with prolonged time to complete the obstacle course but does not correlate with the error frequency: the two transgenic pigs that approached the obstacles more often for the purpose of smelling or licking did not do any errors. Taken together, the results of these behavioural evaluations are consistent with transgenic animals displaying symptoms of visual impairment which they may compensate using other senses.

### Morphological Alterations of Cones in GUCY2D^E837D/R838S^ Transgenic Pigs

**Figure 4:**
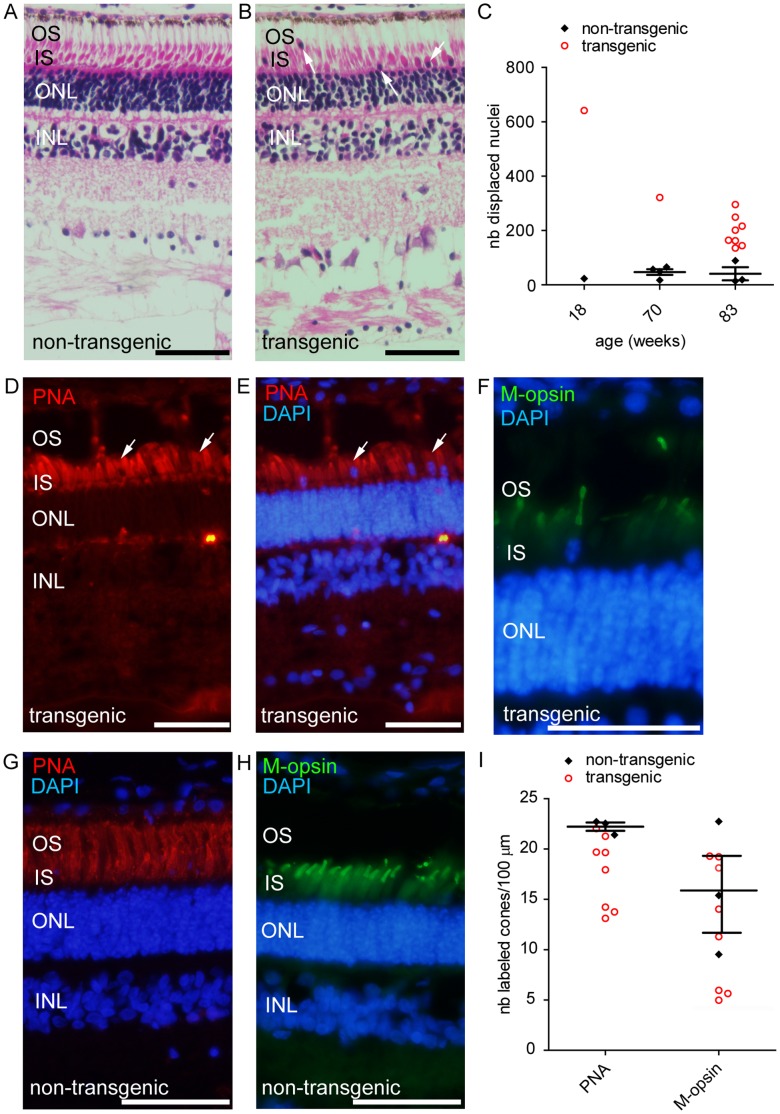
Abnormal retinal morphology in transgenic pigs. (A) Morphological examination of a retina from a control animal reveals retinal layers: outer segments (OS), inner segment of photoreceptors (IS), photoreceptor nuclei (ONL). (B) Displaced nuclei were observed in the outer segment layer in transgenic retina (arrows). (C) Quantification of the number of displaced nuclei in transgenic and control animals. (D,E,F) Immunolabeling for specific cone markers PNA and M-opsin in transgenic retina identified most of these displaced cells as cones. (G,H) Immunolabeling for specific cone markers PNA and M-opsin in control retina. (I) Quantitation of relative density of displaced cones as determined by PNA or M-opsin labeling across 100 µm on the section. OS: outer segment; IS: inner segment; ONL: outernuclear layer; PNA: peanut agglutinin; M-opsin: medium wavelength opsin; nb: number. Scale bar in A, B and D to F represents 50 µm.

**Figure 5:**
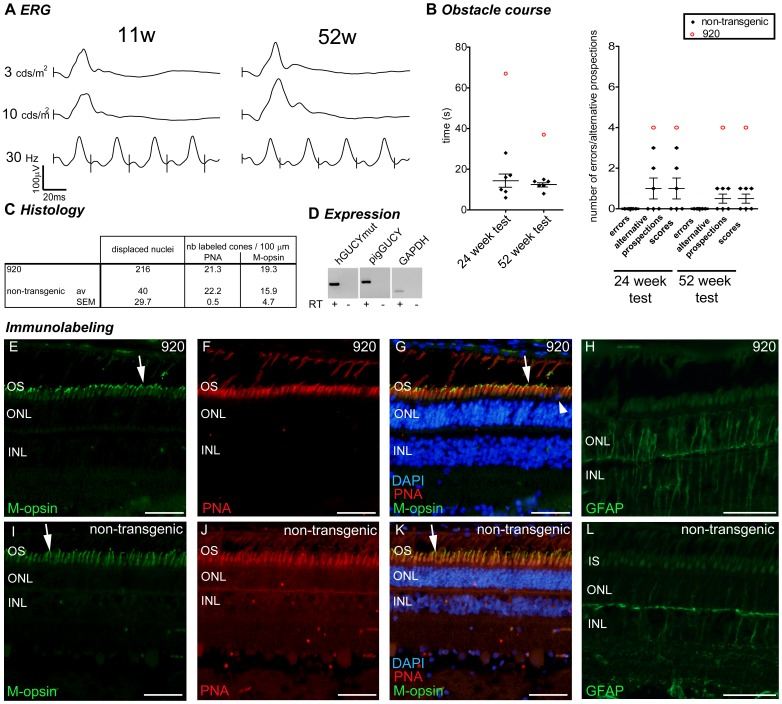
Case reports: pig 920. Grouped data collected for the transgenic pig 920 (4 integrated copies). (A) Photopic ERG (single flash 3 and 10 cds/m^2^ and 30 Hz flicker) at 11 and 52 weeks. (B) Behavioural observation for pig 920 (red opened circle) compared to non-transgenic control animals (black lozenges, mean and SEM depicted). (C) Histological quantification compared to the mean ± SEM of the non-transgenic controls. (D) RT-PCR analysis of transgene, endogenous GUCY2D and GAPDH gene expression in the retina. (E–L) Immunolabeling for M-opsin (E,I), PNA (F,J) and merged picture (G,K), and GFAP (H,I) in the central region of the retina of pig 920 (E–H) and a non-transgenic control (I–L). Arrows in E, G, I and K show examples of M-opsin positive outersegment, arrowhead in G shows a displaced nucleus. W: weeks of age; OS: outer segment; ONL: outer nuclear layer (photoreceptor nuclei); INL: inner nuclear layer (interneuron nuclei); IS: inner segment; M-opsin: M-opsin antibody in green; PNA: peanut agglutinin in red; DAPI: dapi counterstaining in blue; GFAP: Glial fibrillary acidic protein in green; S-opsin: short wavelength opsin in green. Scale bar in E–L represents 50 µm.

**Figure 6:**
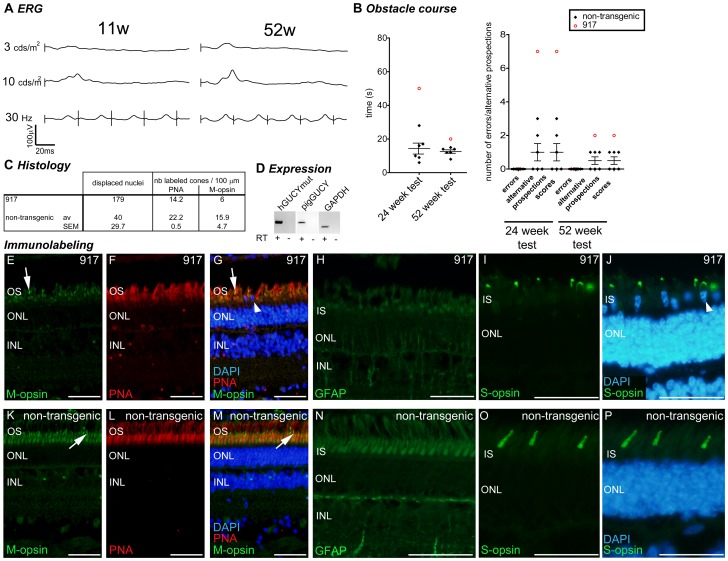
Case reports: pig 917. Grouped data for the transgenic pig 917 (3 integrated copies). (A) Photopic ERG (single flash 3 and 10 cds/m^2^ and 30 Hz flicker) at 11 and 52 weeks. (B) Behavioural observation for pig 917 (red opened circle) compared to non-transgenic control animals (black lozenges, mean and SEM depicted). (C) Histological quantification compared to the mean ± SEM of the non-transgenic controls. (D) RT-PCR analysis of transgene, endogenous GUCY2D and GAPDH gene expression in the retina. (E–P) Immunolabeling for M-opsin (E,I), PNA (F,J) and merged picture (G,K), GFAP (H,I) and S-opsin (I,J,O,P) in the central region of the retina of pig 917 (E–J) and a non-transgenic control (K–P). Arrows in E, G, K and M show examples of M-opsin positive outersegment, arrowhead in G shows displaced nuclei, arrowhead in J shows a displaced nucleus in a S-opsin positive cell. W: weeks of age; OS: outer segment; ONL: outer nuclear layer (photoreceptor nuclei); INL: inner nuclear layer (interneuron nuclei); IS: inner segment; M-opsin: M-opsin antibody in green; PNA: peanut agglutinin in red; DAPI: dapi counterstaining in blue; GFAP: Glial fibrillary acidic protein in green; S-opsin: short wavelength opsin in green. Scale bar in E–J represents 50 µm.

To verify whether the alteration of visual function observed was associated with retinal morphology changes, *in vivo* imaging was performed on anesthetized pigs by spectral domain optical coherence tomography (SDOCT) at 24 and 52 weeks of age. This examination did not reveal severe modifications of the retinal morphology of transgenic pigs when compared to control animals (Figure S2). Pigs were sacrificed at different time points (18, 70 and 83 weeks) to enable further histological analysis of the retina. Consistently with SDOCT images, we didn’t notice any major loss of structure at the gross morphological level (Figure 4A,B). Some transgenic animals however appeared to have significant retinal modifications when analysed at a cellular scale, including an alteration of the compact cellular organisation characteristic of the photoreceptor outer nuclear layer (ONL) and the interneuron inner nuclear layer (INL) (Figure 4A,B). We also observed Müller cell activation, revealed though GFAP immunoreactivity in the ONL which may explain the slight loss of ONL compaction (Figure 5H). This gliosis phenomenon is a typical feature of diseased retina and was not observed in control animals (Figure 5L, Figure 6N). More interestingly, in all transgenic animals, even at the earliest time point analysed (18 weeks), displaced nuclei were observed in the outer segment (OS) layer, (Figure 4A,B). While such displaced nuclei could be detected in both transgenic and control animals, they were significantly increased in the transgenic group for all the ages investigated (p<0.01, Figure 4C). Most of these ectopic nuclei colocalised with cone markers: components of the cone extracellular matrix (recognised by PNA) and cone photopigments (M-opsin or S-opsin) (Figure 4D–F; Figure 5E–G, Figure 6E–G,I,J). Further quantification of M-opsin and PNA positive cells revealed decreased expression of these proteins in the OS of some transgenic animals (Figure 4I), suggesting an alteration of cone health. This decrease was correlated to a diminution of cone OS number determined by morphological observation of paraffin sections (pigs 907, 917, 918 mean ±SEM: 14±1 OS/100 µm, control group mean ±SEM: 18±1 OS/100 µm, n = 3, *p* = 0.02). Results of histological analysis thus reveal subtle signs of retinal alteration in the transgenic group.

### Variability of the Phenotype of GUCY2D^E837D/R838S^ Transgenic Pigs: Case Report

**Figure 7:**
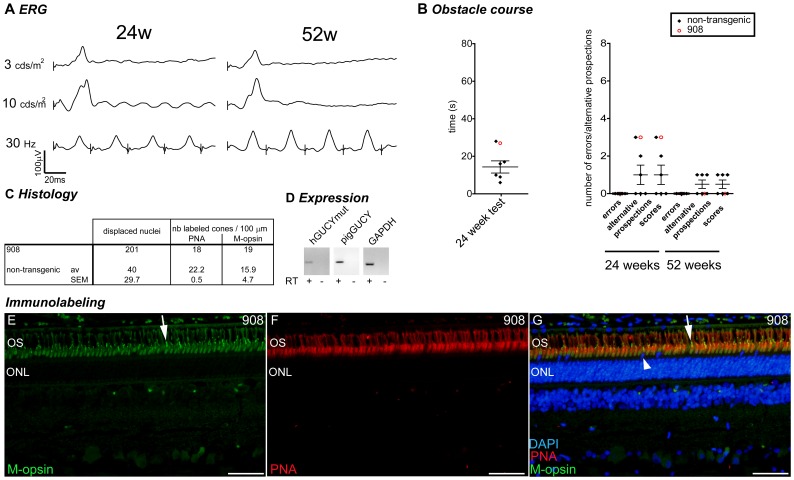
Case report: pig 908. Grouped data for the transgenic pig 908 (2 integrated copies). (A) Photopic ERG (single flash 3 and 10 cds/m^2^ and 30 Hz flicker) at 11 and 52 weeks. (B) Behavioural observation for pig 908 (red opened circle) compared to non-transgenic control animals (black lozenges, mean and SEM depicted). For technical reasons, the time to reach the end of the obstacle course has not been measured at 52 weeks. (C) Histological quantification compared to the mean ± SEM of the non-transgenic controls. (D) RT-PCR analysis of transgene, endogenous GUCY2D and GAPDH gene expression in the retina. (E–G) Immunolabeling for M-opsin (E), PNA (F) and merged picture (G) in the central region of the retina of pig 908. Arrows in E and G show examples of M-opsin positive outersegment, arrowhead in G shows a displaced nucleus. W: weeks of age; OS: outer segment; ONL: outer nuclear layer (photoreceptor nuclei); INL: inner nuclear layer (interneuron nuclei); IS: inner segment; M-opsin: M-opsin antibody in green; PNA: peanut agglutinin in red; DAPI: dapi counterstaining in blue; GFAP: Glial fibrillary acidic protein in green; S-opsin: short wavelength opsin in green. Scale bar in E–G represents 50 µm.

Taken together, the results of characterization of the transgenic animals reveal retinal alteration of the transgenic animals when compared to controls. Of note, an important phenotypic heterogeneity is observed among the transgenic group that likely reflects the heterogeneity inherent to the generation of outbred transgenic animals associated with random insertion of a promoter-transgene cassette. In essence each animal in the transgenic cohort is unique as a consequence of its genetic background, the variation in the natural level of its endogenous GUCY2D gene associated with variable expression of the transgene, whereby both copy number and position effect can influence expression levels. We selected particular transgenic pigs that are described hereafter in a case report manner to further illustrate this diversity: pigs 908, 920 and 917, respectively genotyped with 2, 4 and 3 integrated copies of the vectors; transgene expression was confirmed in each one *post mortem* (83 weeks, Figure 7D, 5D, 6D).

Two animals were particularly poorly affected during the course of the experimentations. As an illustration, results of pig 908 phenotyping are recapitulated in Figure 7. This animal maintained relatively normal features, except an increase in the number of displaced nuclei (Figure 7C). Longer follow up of this animal would have been required to determine whether it was developing a milder or delayed form of the disease. This clearly contrasts with several other transgenic animals that displayed both earlier onset and more significant progression throughout the course of the reported observations.

Pig 920 maintained normal retinal function up to 52 weeks regarding ERG recordings but progressed slower through the obstacle course and with more difficulties than non-transgenic animals (Figure 5A–B). On the histological level, at 83 weeks of age, despite normal cone labelling (PNA, M-opsin markers) (Figure 5E–G), we observed increased displaced nuclei (Figure 5C) and GFAP staining in the ONL (Figure 5H,L) revealing Müller cell activation and retinal damage. Thus, retinal expression of the transgene GUCY2D^E837D/R838S^ correlates with abnormal retinal morphology and progression through obstacles but didn’t lead to a severe phenotype by 52 weeks of age.

Pig 917 showed a more pronounced phenotype with retinal activity being severely reduced by 11 weeks of age (Figure 6B). This phenotype was maintained throughout the experiment and no further decrease of the ERG response was observed at 52 weeks (Figure 6A). Behavioural observations suggest that useful vision was affected, with this animal taking more time to finish the obstacle course and conducting many more alternative prospections than did controls (Figure 6B). In parallel, an increased number of displaced nuclei and a reduced number of PNA- or M-opsin-labeled cones were observed at 83 weeks of age (Figure 6C,E-G). Increased GFAP staining was also noticed in the ONL, although less pronounced than in pig 920 (Figure 6H). Thus, expression of the GUCY2D^E837D/R838S^ transgene in pig 917 is associated with reduced ERG response, reduced visual acuity and altered retinal morphology.

## Discussion

### GUCY2D^E837D/R838S^ Transgenic Pigs as a Relevant Model to Study Cone Dystrophies

A transgenic pig line generated through SCNT has previously been used to study rod diseases [37]. Our study further shows that cone diseases can be investigated and monitored in the pig retina by functional and histological analysis. Until now, animal models to study cone biology were poor. Genetic studies in humans have established a strong correlation between a severe cone–rod dystrophy and the GUCY2D^E837D/R838S^ mutations suggesting that this mutation affects cone metabolism [17]–[19], [38]. Recently, Collery *et al.* [39] described a zebrafish model, generated using the same CORD6 double mutation described above (GUCY2D^E837D/R838S^). The animals displayed altered retinal structure with reduction of the number of photoreceptor nuclei as well as reduction of some specific photoreceptor markers. Retinal modifications were observed as early as at the larvae stage but the progressive nature of the symptoms was not easily appreciated, probably because of the retinal regenerating capacity in these animals. Surprisingly, these structural defects were not associated with alteration of the visual function, as measured by optokinetic response. In contrast, the GUCY2D^E837D/R838S^ transgenic pigs we describe displayed a modified behavioral phenotype, a reduced retinal function as measured by ERGs and histological evidences of retinal alterations, features that are closer to the human disease than the zebrafish model.

ERG, recorded using the human protocol (ISCEV), revealed significant differences between transgenic and non-transgenic animals as early as 11 weeks. Simple visual behavioral tests applied to assess the useful vision of the pigs revealed clear changes between the most affected animals and the controls and evidenced visual impairment leading to reduced vision in transgenic pigs at 24 and 52 weeks. The test built for 11 weeks animals was probably not stringent enough to discriminate mild vision impairments, but it suggests that no dramatic visual impairment occurs at this early age. Indeed, in the first visual test settings, the distance to cover was short and doors to cross might have been too easy to detect. The correlation between low ERG and impaired vision-conducted behavior is not obvious. Some animals with low ERG have low vision while others navigate well through the obstacles. Conversely, animals with nearly normal ERG exhibit impaired navigation capacity. One may hypothesize that animals with slow disease progression could display a degree of adaptation, whereby they learned to cope with reduced visual acuity over time. Alternatively, it may be that degeneration within the cone-enriched horizontal streak is not homogeneous, resulting in regions of impaired vision rather than a more general diminution of visual acuity – in this scenario specific visual impairment would not necessarily correlate directly with gross ERG reduction because partial loss can be masked in the global response recorded by ERG.

Similarly, progressive visual impairment is commonly observed in patients affected by cone dystrophies, such as CORD6, with disease onset spanning the first decade [18], [38], [40], [41]. This decrease of the visual acuity is the first symptom occurring in patients followed by impairment in color discrimination in a later phase of the disease. Recent studies report learning ability of pigs [42]–[46] allowing in the future the investigation of such phenotypes in pig through more complex behavioral tests. Retinal structure was assessed by *in vivo* imaging (SDOCT) and *post-mortem* histology. The only available description of CORD6 patient’s retina is the report of Kim *et al.* [40] describing the use of SDOCT to visualize photoreceptor abnormalities in a 13-year old patient bearing a GUCY2D^R838H^ mutation. This child had experienced progressive loss of vision in both eyes over the preceding 3 years, with reduced ERG. OCT examination revealed several abnormalities of the retina, including thinning at the fovea of each eye as well as thinning of the RPE in the central retina and structural alteration of outer-segments. Although such alterations were not observed in the transgenic animals through either OCT or histology, we report remodeling consistent with retinal stress: decreased number of the PNA- and M-opsin positive cone cells, decreased number of cone outer-segments, increased gliosis and increased number of cone nuclei displaced in the outer-segment layer. Ectopic nuclei are a hallmark of certain cone dystrophies, as shown in the XLPRA dogs bearing mutations in the RPGR photoreceptor ciliary protein [47] as well as in the model of slow progressive retinopathy in Shetland sheepdogs [48]. This result suggests that the GUCY2D^E837D/R838S^ transgenic pigs show early signs of the degeneration process, which has not previously been described in patients.

Taken altogether, our results show that the features of the cohort of GUCY2D^E837D/R838S^ transgenic pigs recapitulate well the features of CORD6 human patients [49]–[51]. Further characterization of this model will contribute to a better understanding of the physiopathology of CORD, thereby supplementing ongoing investigations in human patients. Most interestingly, the increased presence of displaced nuclei in the segment layers appears to be a common feature of cone dystrophy and could be an early marker of the disease.

### Pig Lentiviral Transgenesis to Model Diseases with Phenotypic Heterogeneity

Beyond the ophthalmology field, pigs have many anatomical and physiological similarities to humans, including their cardiovascular system, omnivorous gastrointestinal tracts and immune system. They are thus recognized as excellent models for a variety of diseases and are appropriate for development of new surgical, endoscopic and delivery techniques. Together with the recent advances in the fields of genetic modification of large species, pig is particularly suited for developing new transgenic animal models.

The present study shows that lentiviral transgenesis in pig allows the easy and rapid generation of a large number of transgenic founder animals expressing a dominant-negative allele, without the need to establish a colony. Because gilts may deliver 8 to 14 piglets with a gestation time of only 16 weeks, investigators may envisage generating as many founders as needed for their preclinical investigations in a relatively short period of time. For instance, in this study, only two gilts were necessary to generate 13 transgenic animals and their non-transgenic littermate controls.

In addition to efficiency, another advantage of the approach we used in this study is the heterogeneity it generates. Indeed, altered retinal morphology coupled with reduced function are observed among GUCY2D^E837D/R838S^ animals with a spectrum of severity that mimics the heterogeneity observed in patients, allowing to perform individual case studies in the transgenic animals. This diversity is likely due to a combination of transgene expression level as a function of copy number and integration site, coupled with the variable genetic background of the cohort (outbred siblings and half-siblings). This is one of the strengths of this approach as such variety is reminiscent of the variability generated by a specific mutation type in different human patients, showing incomplete penetrance or variability of symptom severity. This phenomenon has been described for many human conditions, including CORD6 patients which are differently affected, either regarding the kinetics or the severity of the symptoms [18], [38], [41]. This has also been described in animal models through the comparison of inbred strains of mice [52]–[59]. Although inbred strains have been invaluable tools for deciphering many fundamental mechanisms, understanding human conditions and developing efficient and safe treatment should take into account natural heterogeneity. In the context of our study, it enabled to point out the increased presence of displaced nuclei and gliosis as common feature of the disease. Thus, the diversity of phenotypes generated by our approach facilitate the identification of the common deleterious effects associated with the studied mutation and may as well contribute to the identification of cofactors that positively or negatively impact on the pathological process. Although introducing heterogeneity in preclinical investigations might be seen at first sight as increasing the scattering of the results and hampering their interpretation, heterogeneity may be sought in order to better understand the human pathology, as illustrated in the present study.

## Supporting Information

Figure S1 **Obstacle courses.** (A) Photograph of the maze used when pigs were 11 weeks of age. (B) Photograph of obstacle course used to assess animals at 24 weeks of age. (C) Photograph of obstacle course used to assess animals at 52 weeks of age.
(TIF)

Figure S2 **Example of** ***in vivo*** **imaging of pigs.** OCT Scans of the central region of a non-transgenic control (A) and two examples of transgenic animals (B,C) at 24 weeks show the presence of both photoreceptor nuclei (ONL) and outer segments (OS). Vertical bar indicate the thickness of layers representative of the outer nuclear layer (ONL) or outer segments (OS).
(TIF)

Movie S1 **Examples of a control pig and a transgenic pig navigating through the obstacle course at 24 weeks of age.**
(MP4)
