# Supplementary figures and images for: Rapid Cohort Generation and Analysis of Disease Spectrum of Large Animal Model of Cone Dystrophy
Source: PLoS One. 2013 Aug 19;8(8):e71363. doi: 10.1371/journal.pone.0071363 (PMC3747164; doi:10.1371/journal.pone.0071363)

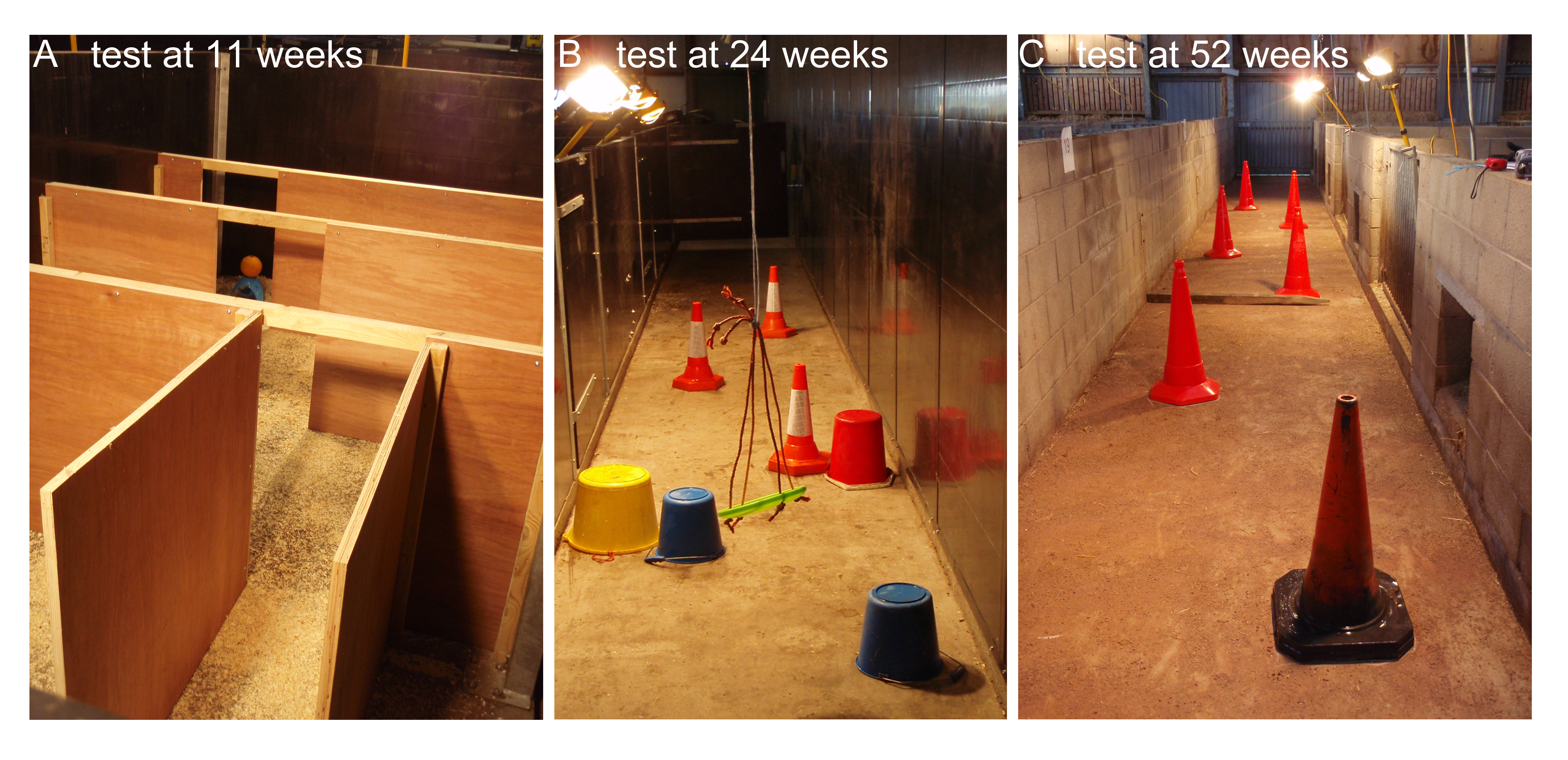

Supplement: Figure S1 — Obstacle courses. (A) Photograph of the maze used when pigs were 11 weeks of age. (B) Photograph of obstacle course used to assess animals at 24 weeks of age. (C) Photograph of obstacle course used to assess animals at 52 weeks of age. (TIF) [file pone.0071363.s001.tif]

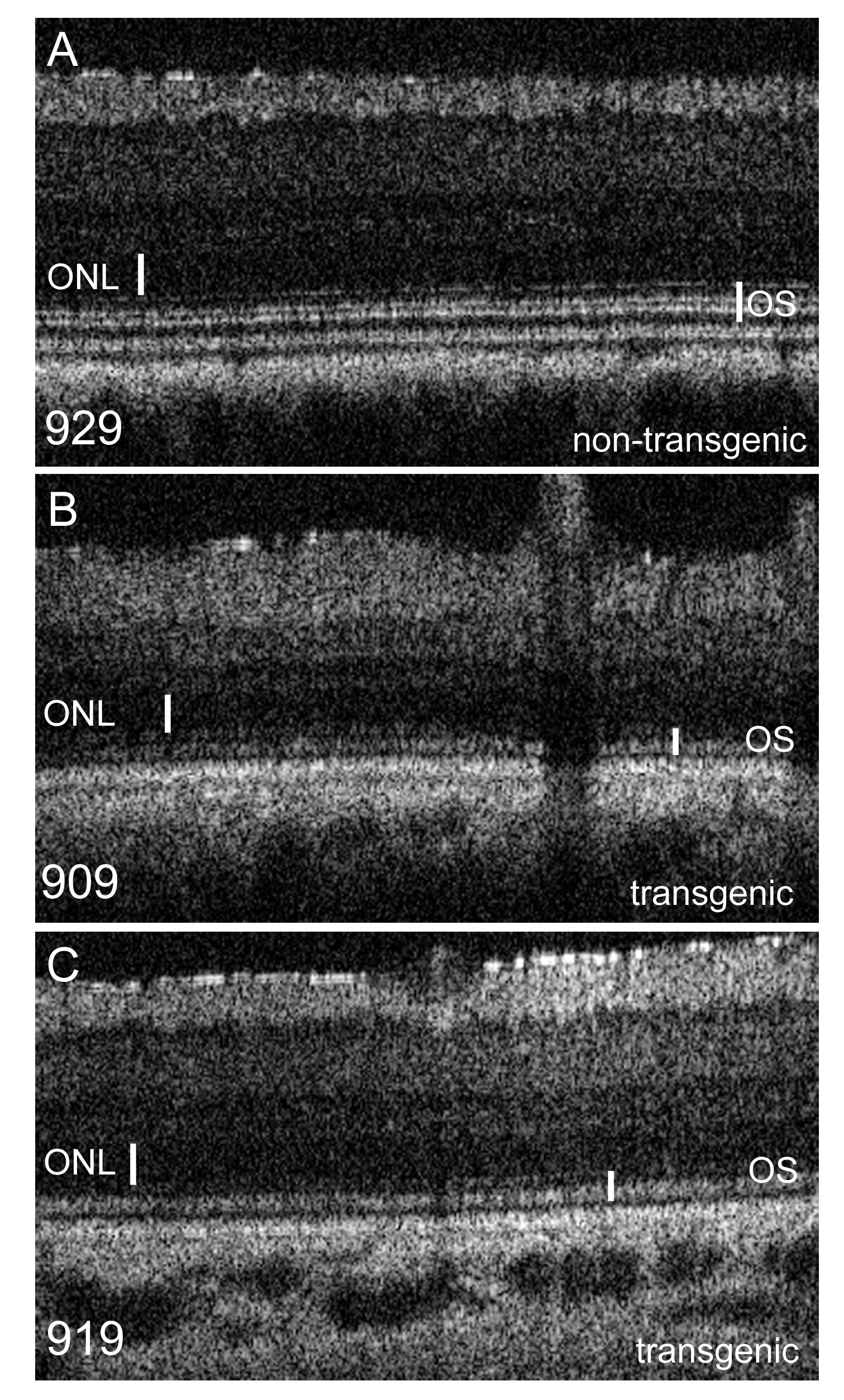

Supplement: Figure S2 — Example of in vivo imaging of pigs. OCT Scans of the central region of a non-transgenic control (A) and two examples of transgenic animals (B,C) at 24 weeks show the presence of both photoreceptor nuclei (ONL) and outer segments (OS). Vertical bar indicate the thickness of layers representative of the outer nuclear layer (ONL) or outer segments (OS). (TIF) [file pone.0071363.s002.tif]
